# Editorial

**Published:** 1868-07

**Authors:** 


### Editorial.

The record of Proceedings of the Illinois State Medical Society occupy so much more space than we expected, that we are obliged to omit from the present number both the clinical report and some book notices. They will appear in our next issue.

Chicago Medical College.—We have received the Tenth Annual Announcement of this Institution, by which the public are informed of some important changes. The two important chairs of Chemistry have been filled by the appointment of Prof. Wheeler, a gentleman of the highest order of attainments in that department of science. He has promptly established a laboratory for instruction in practical chemistry, which will give to the medical students advantages equal to those heretofore enjoyed only at Ann Arbor and Cambridge.

The clinical advantages have also been made more extensive by the appointment of Professors Johnson, Byford, and Bevan in the active staff of attendants in the County Hospital. This, added to the previous clinical advantages of Mercy Hospital, renders the means for bedside instruction equal to any institution in this or other countries.

But the item to which we wish to call the attention of our readers most particularly, is the full provision for the improved system of Medical College Instruction recommended by the Convention of Delegates from Medical Colleges, held at Cincinnati, in May, 1867. Nearly all the State Medical Societies in the North-West, as well as the profession at large, have emphatically endorsed this plan, and called earnestly on the colleges for its adoption in practice. They have now an opportunity to show the sincerity of their recommendations, by properly directing the students under their charge or influence.

---

Money Receipts to June 29th.—Drs. A. D. Andrews, $5; James Culbertson, 3; Stacy Hemenway, 5; H. C. Newkirk, 1.50; Thos. W. Howes, 3; W. D. Sterling, 3; B. Wilson, 3; F. K. Bailey, 2; James W. Mill, 1; George A. Bardwell, 3; H. P. Oggell, 3; J. B. Newman, 3; V. H. Coffman, 5; G. P. Martin, 3; Wm. Dougall, 1.50.

---

A Hint for Women.—Mrs. Caroline H. Dall says: "I have looked in women's faces to see what marks their lives have left, and I tell you that it is a simple fact that women who habitually prevent impregnation grow cold, debased, unlovely in their expression; and that those who resort to abortion become sharp, irritable, and ungenial, everything in short that we mean by unmotherly."

MORTALITY REPORT FOR THE MONTH OF MAY:—

CAUSES OF DEATH.

| | | | | | |
|---|---|---|---|---|---|
| Accident, drowned, | 4 | Dropsy | 3 | Manslaughter, | 1 |
| " railroad, | 1 | Dysentery, | 2 | Measles | 5 |
| " machinery, | 1 | Eczema, congenital, | 1 | Metritis, puerperal, | 1 |
| " revolver, | 1 | Enteritis, chronic | 1 | Meningitis | 2 |
| Angina | 1 | Erysipelas, | 1 | " cerebro-spinal, | 4 |
| Apoplexy | 6 | " phlegmonous | 1 | " tuberculosis, | 1 |
| Asphyxia | 1 | Exastisis of 2d cervical | | Myelitis | 1 |
| Birth, premature | 13 | vertebra resulting in | | Nephritis acuto, result- | |
| " still | 28 | hemiplegia, | 1 | ing in uræmic convul- | |
| " tedious | 1 | Fever, puerperal | 1 | sions, | 1 |
| Brain, compres. at birth | 1 | " scarlet | 13 | Old Age | 2 |
| " congestion of | 4 | " typhoid | 5 | " and dropsy, | 1 |
| " disease of | 1 | " typhus | 1 | Paraphlegia, traumatic, | 1 |
| " dropsy of | 1 | Gastritis | 1 | Paralysis | 1 |
| " inflammation | 7 | " chronic | 1 | " general, | 1 |
| " injury by blow, | 1 | Heart, disease of | 1 | Peritonitis | 2 |
| " softening of, | 1 | " organic disease | 1 | Phthisis pulmonalisis | 36 |
| Bright's disease | 2 | Hemorrhage, internal, | 1 | " " and chorea, | 1 |
| Bronchitis | 5 | Hydrothorax, | 1 | " laryngeal, | 1 |
| " capillary | 1 | " fol'ing phthi- | | Phrenitis, | 3 |
| " chronic | 2 | sis pulmonalis, | 1 | Pneumonia, | 18 |
| Bowels, inflammation | 3 | Hydrocephalus | 9 | " and pericar- | |
| " " & old age | 1 | " acute, | 3 | ditis with hypertrophy | |
| Cancer | 1 | Intestines, intussuscep- | | of left and fatty degen- | |
| " of uterus, | 1 | tion and ulceration, | 1 | eration of right ventricle | 1 |
| " of breast, | 2 | Invagination ileus, | 1 | Rheumatism, inflam'ory | 1 |
| " of neck & throat, | 1 | Infanticide, by mother, | 1 | " back & spine, | 1 |
| Convulsions | 36 | Inanition | 13 | Skull, fracture of, | 1 |
| " puerperal, | 1 | Indigestion and old age, | 1 | Small-pox | 12 |
| Compound comminuted | | Inflammation caused by | | " complicated | |
| fracture of right femur | 1 | constipation of bow'ls, | 1 | with pneumonia | 1 |
| Chlorosis | 1 | Insufficiency of aortic | | Stomach, inflammation | 2 |
| Cholera infantum | 1 | semilunar valves, | 1 | Suicide | 3 |
| Croup | 4 | Insanity, | 1 | Scrofula | 1 |
| " membranous, | 2 | " resulting from | | Tabes mesenterica, | 7 |
| Cyanosis | 1 | epilepsy, | 1 | Tetanus traumaticus, | 1 |
| Debility | 5 | Intemperance | 1 | Teething | 10 |
| Delirium tremens | 1 | Jaundice | 1 | " and convulsions, | 1 |
| Deficient nutrition, | 1 | Laryngitis, tubercular, | 2 | Unknown | 1 |
| Diphtheria | 4 | Liver, cirrhosis of | 1 | Uterus, rupture of | 1 |
| Diarrhœa | 6 | Lungs, congestion of | 3 | | |
| " chronic, | 1 | " abscess of | 1 | Total | 321 |

Deaths in May, 1868, 321 | Deaths in May, 1867, 241 | Increase, 80

Deaths in April, 1868, 305 | Increase, 16

AGES.

| | | | | | |
|---|---|---|---|---|---|
| Under 5 | 167 | 40 to 50 | 23 | 90 to 100 | 0 |
| 5 to 10 | 22 | 50 to 60 | 17 | 100 to 110 | 0 |
| 10 to 20 | 16 | 60 to 70 | 7 | Unknown | 1 |
| 20 to 30 | 29 | 70 to 80 | 9 | | |
| 30 to 40 | 27 | 80 to 90 | 3 | Total | 321 |

| | | |
|---|---|---|
| Males, 178 | Females, 143 | Total, 321 |
| Single, 238 | Married, 83 | Total, 321 |
| White, 310 | Colored, 11 | Total, 321 |

NATIVITY.

| | | | | | |
|---|---|---|---|---|---|
| Chicago | 126 | England | 7 | Scotland | 1 |
| Other parts U. S. | 62 | Germany | 47 | Sweden | 11 |
| Bohemia | 8 | Holland | 3 | Switzerland | 2 |
| Canada | 3 | Italy | 1 | Unknown | 3 |
| Denmark | 0 | Ireland | 38 | | |
| France | 2 | Norway | 7 | Total | 321 |

DEATHS BY SMALL-POX.

For the Month of May, 1868.

| | | | |
|---|---|---|---|
| 6th Ward | 1 | 13th Ward | 4 |
| 7th Ward | 1 | 15th Ward | 2 |
| 11th Ward | 1 | Lake Hospital | 1 |
| 12th Ward | 3 | | |
| | | Total, | 13 |

MORTALITY BY WARDS FOR THE MONTH.

| Ward. | Mortality. | Pop. in 1866. | One death in | | Ward. | Mortality. | Pop. in 1866. | One death in | |
|---|---|---|---|---|---|---|---|---|---|
| 1 | 3 | 9,648 | 3,216 | | 14 | 19 | 12,108 | 637 | 1-4 |
| 2 | 15 | 12,985 | 865 | 2-3 | 15 | 32 | 15,766 | 491 | 1-8 |
| 3 | 16 | 15,738 | 983 | 5-8 | 16 | 11 | 14,912 | 1,355 | 7-11 |
| 4 | 19 | 10,884 | 572 | 4-5 | County hosp. | 14 | | | |
| 5 | 23 | 9,610 | 409 | 1-8 | Unknown, | 1 | | | |
| 6 | 19 | 10,680 | 556 | 8-10 | Alexian Bros. | 1 | | | |
| 7 | 21 | 18,755 | 893 | 1-10 | Home of the Friendless, | 2 | | | |
| 8 | 16 | 10,429 | 651 | 6-8 | Soldier's hom. | 1 | | | |
| 9 | 21 | 13,940 | 663 | 1-5 | Mercy hos., | 4 | | | |
| 10 | 11 | 11,416 | 1,037 | 9-11 | St. Luke's hos. | 2 | | | |
| 11 | 13 | 12,924 | 994 | 2-13 | Orphan asyl. | 8 | | | |
| 12 | 28 | 12,695 | 453 | 3-7 | Lake Hosp., | 1 | | | |
| 13 | 20 | 8,188 | 409 | 2-5 | | | | | |
| Total, | | | | | | 321 | | | |

THE LATE DR. R. T. RICHARDS.—At the annual meeting of the De Witt County Medical Society, held on the 11th day of May, 1868, Dr. C. Goodbrake, from the committee appointed to draft resolutions expressive of the sense of this Society on the death of our late colleague, Dr. Richards, reported the following preamble and resolutions, which were unanimously adopted:

*Whereas*, It has pleased God, in His wise Providence, to remove from our midst and from a field of great professional usefulness, our highly esteemed friend and co-laborer in the science and practice of medicine, Dr. Rolla T. Richards, who died at Santa Anna, on the 12th day of March, 1868; be it therefore

*Resolved*, That we deeply feel the loss of our deceased friend and brother, who, by his upright, moral conduct, and gentlemanly deportment, had endeared himself to all with whom he became associated.

*Resolved*, That, in his death, the profession is deprived of one of its most zealous and devoted members, and the community of a *good physician*, and one of her best and most patriotic citizens.

*Resolved*, That we feel a sincere sympathy for his widow in her great affliction, and can only refer her, with unfeigned confidence, to Him who has promised to be the widow's God.

*Resolved*, That the foregoing preamble and resolutions be spread upon the records of the Society; an attested copy sent to the widow of the deceased, and one to each of the medical journals published in Chicago, for publication.

C. GOODBRAKE,<br>J. WRIGHT,<br>Z. H. MADDEN. } *Committee.*

J. A. EDMISTON, M.D., *Pres't.*

C. GOODBRAKE, M.D., *Sec'y.*

---

1504 WALNUT STREET, PHILADELPHIA, *May 26, 1868.*

*Dear Sir:*—I am instructed by the Committee of Publication of the American Medical Association to call your attention to the following amendment to the "Plan of Organization," adopted at the late meeting held in Washington City, and to request you to forward the amount named to the Treasurer, 1303 Arch Street, on or before the 1st day of July, proximo, in order that the requisite number of copies of the forthcoming edition may be printed without delay. Very respectfully yours,

FRANCIS G. SMITH, JR., *Chairman.*

"The sum of $5 shall be assessed annually upon each delegate to the sessions of the Association, as well as upon each of its Permanent Members, whether attending or not, for the purpose of raising a fund to defray the necessary expenses of the Association, and for printing the Transactions. The payment of this sum shall be required of the delegates and members in attendance upon the sessions of the Association previously to their taking seats and participating in the business of the session. Permanent Members not attending shall forward their yearly dues to the Treasurer, and thereby shall be entitled to receive a copy of the Transactions, the same as delegates."

---

### O'REILLY PRIZE.

Dr. JOHN O'REILLY, of New York, having offered, through the N.Y. Academy of Medicine, a Prize of Six Hundred Dollars for an Essay on the Physiology and Pathology of the Sympathetic or Ganglionic Nervous System, the Committee of Award, appointed by the Council of the Academy, have adopted, with the concurrence of the Council, the following regulations:

I. The competing essays shall be sent in to the Chairman of the Committee, Prof. J. C. Dalton, M.D., No. 101 East Twenty-Third Street, New York, on or before the First day of March, 1869.

II. Each Essay shall be marked with some distinguishing device or motto, and accompanied by a sealed envelope bearing the same device or motto, and containing the name and address of the writer.

III. The Essay selected by the Committee shall be transmitted by them, together with its accompanying envelope, to the Council of the N.Y. Academy of Medicine, under whose direction the envelope shall be opened and the name of the writer announced at the first meeting of the Academy in May, 1869.

IV. This Prize is open for universal competition.

V. The Committee have a right to reject whatever does not come up to a proper standard of merit.

ALFRED C. POST, M.D., *Pres't of the Acad'y,*
On behalf of the Council.

*Committee of Awards.*—J. C. Dalton, M.D., Professor of Physiology in the College of Physicians and Surgeons, New York; A. Flint, Jr., M.D., Professor of Physiology in Bellevue Hospital Medical College, New York; Alfred L. Loomis, Professor of the Institutes and Practice of Medicine in University Medical College, New York.

*New York, December, 1867.*

